# Choosing a skull clearing technique for chronic mesoscopic optical imaging in awake mice

**DOI:** 10.1117/1.NPh.13.2.025006

**Published:** 2026-03-24

**Authors:** Bengisu Solgun, Buket Dönmez-Demir, Aslıhan Bahadır-Varol, Hülya Karataş, Şefik Evren Erdener

**Affiliations:** Hacettepe University, Institute of Neurological Sciences and Psychiatry, Ankara, Turkey

**Keywords:** mouse skull, optical clearing, optical transparent window, cortical spreading depression, laser speckle contrast imaging, intrinsic optical signal imaging

## Abstract

**Significance:**

Transcranial imaging windows are crucial for *in vivo* imaging in rodents. Although conventional methods such as open-skull and thinned-skull windows have been widely utilized, minimally invasive skull clearing methods have recently been developed and have yet to be comprehensively evaluated for chronic mesoscopic imaging in awake mice.

**Aim:**

We aimed to methodically compare and modify current topical skull optical clearing methods in search of an optimal technique for chronic awake widefield imaging.

**Approach:**

We used widefield intrinsic optical-signal imaging and laser speckle contrast imaging to investigate the applicability of these methods.

**Results:**

Application of a UV-curable optical adhesive, NOA61 both as a monotreatment and in combination with EDTA, proved suitable for long-term awake mesoscopic imaging, providing long-term global transparency. The previously described Triple-S method led to inflammatory-like reaction and/or opacification of the window over subsequent days. Cyanoacrylate or methyl-methacrylate (Metabond-equivalent) treatments, on the other hand, led to drying artifacts, occluding parts of the window. Therefore, these latter methods made awake bihemispheric imaging impractical. EDTA-NOA61 combination was then tested for documenting interhemispheric connectivity changes following optogenetic triggering of cortical spreading depolarizations.

**Conclusions:**

We discussed the advantages and drawbacks of previous skull clearing methods and identified suitable methods for awake mesoscopic chronic imaging. These findings highlight the importance of minimally invasive skull optical clearing methods for widefield use in awake mice.

## 1 Introduction

Longitudinal *in vivo* imaging of the brain with state-of-the-art technologies allows the capture of neuronal activities and their hemodynamic integration across different brain regions in living animals, especially rodents, providing invaluable data for neuroscience research. The natural opacity of the mouse skull is a well-known challenge for optical access to the brain, for both means of imaging and optogenetic stimulation. The classical solution, thinning or removing a piece of skull for optical access, is now revealed to be injurious to the cerebral cortex and neurovascular structures, especially in the acute setting, and confounders introduced by anesthesia contribute to these disadvantages of acute craniotomies.^1^^–^^6^ Therefore, the implementation of chronic cranial windows that allow repeated imaging over time, including windows in awake mice, is increasingly being utilized.^7^^–^^17^ Although round glass coverslips provide a relatively narrow field of view, there have been advances in bihemispheric large-field mesoscopic window techniques that allow the mapping of multiple functional regions and their network interactions.^18^^,^^19^ However, these large craniotomies can still have unwanted effects due to dural or brain damage or chronic inflammatory changes lasting long-term.^20^ Removal of the skull can also alter pathophysiology significantly as it is now understood that skull immune cells and vasculature are both important elements with direct communications to leptomeningeal structures.^21^^–^^23^

Accordingly, over the last years, there have been introductions of various approaches for transcranial optical imaging via optical clearing of the mouse skull. These techniques eliminate the need for any invasive surgery, even remotely performed, thereby minimizing brain damage or inflammatory changes. Although they provide inferior optical access compared to a glass window, they can still provide data sufficient for widefield imaging tools like intrinsic optical signal imaging (IOSI), calcium imaging, and laser speckle contrast imaging (LSCI), in addition to allowing optogenetic stimulation of the cortex.^24^ Some groups have even used two- and three-photon imaging at superficial cortical layers through transparent skulls.^7^^–^^9^^,^^11^ These methods usually consist of opening the scalp and topical applications of various solutions or adhesives to the skull. There are many strategies reported in the literature, applicable for acute or chronic repeated imaging, reaching differing levels of transparency for varying durations. It is very likely that different techniques can be suitable for different experimental designs. However, a systematic study comparing the performance of multiple techniques for a chronic longitudinal experimental design was, to our knowledge, lacking. As we wanted to implement the most reliable technique optimal for chronic longitudinal intrinsic optical signal and LSCI on a mesoscopic scale in awake mice that also allows optogenetic triggering of cortical spreading depolarizations (CSDs), we reviewed the previously reported optical skull clearing techniques in mice and experimentally compared the performance of five topical skull optical clearing methods.

### 1.1 Previous Strategies on Optical Skull Clearing

The organic component of the skull is composed of collagen, proteins, lipids, and blood cells, whereas the mostly mineralized inorganic component has hydroxyapatite and calcium phosphate.^8^^,^^25^ To “clear” the skull by topical applications and make it light-permeable, removal of collagen, calcium, or fat; obtaining structural homogeneity by the removal of irregularities; and refractive index matching are needed. Previous skull optical clearing techniques have targeted either one or multiple of these requirements. Table 1 summarizes the previous attempts on optical skull clearing identified in published literature.

**Table 1:** Comparison of skull optical clearing techniques.

Method	Primary clearing agent(s)	Additional clearing agent(s)	Solid/Liquid	Imaging method	Measure of transparency	Window size	Chronicity	Awake
**Transparent skull (TS)** Steinzeig et al.^10^ (Similar to Guo et al.^26^)	Cyanoacrylate, acryl powder, methyl methacrylate liquid, transparent nail polish	Window was polished with a hand drill	Solid	Intrinsic optical signal imaging	Average magnitude of the intrinsic signals, ocular dominance indices	Narrow	2 months	No
**Cyanoacrylate** Couto et al.,^27^ (Similar application in Musall et al.^28^)	Cyanoacrylate	-	Solid	Calcium imaging	Widefield images shown for up to 5 months	Bihemispheric	5 months	Yes
**NOA81** Zatka-Haas et al.^29^ (Similar applications in Burgess et al.,^30^ Steinmetz et al.^31^ and Steinmetz et al.^32^)	NOA81	Cyanoacrylate	Solid	Calcium imaging	None	Bihemispheric	Yes, unspecified	Yes
**Metabond** Silasi et al.^12^ (Similar application in Gilad et al.^33^)	C&B-Metabond	-	Solid	Calcium imaging	Contrast value of vessels	Bihemispheric	2 months	Yes
**SOCS** Wang et al.^34^	Laurinol	EDTA, dimethyl sulfoxide, sorbitol, alcohol, glucose and weak alkaline elements	Liquid	Laser speckle contrast imaging, brighfield imaging	Minimum resolution diameter	Bihemispheric	25 min	No
**SOCW** Zhao et al.^9^	EDTA, glycerol	Thinned skull in adult mice	Liquid - needs reclearing prior to imaging	Two-photon imaging, confocal microscopy	Fluorescence intensity, imaging depth	Narrow	21 days with acute reclearing prior to imaging	No
**USOCA** Zhang et al.^7^	S1 (ethanol and urea), S2 (SDBS)	-	Liquid - needs reclearing prior to imaging	Two-photon imaging, laser speckle contrast imaging, hyperspectral imaging, and brighfield imaging at 550nm	Imaging depth, signal intensity, minimum resolvable vascular diameter, contrast to noise ratio (CNR), vessel diameter	Narrow (Bihemispheric windows evaluated in the acute period)	6 months, with acute reclearing prior to imaging	No
**TIS window** Li et al.^8^	S1, S2, S3 (UV-curable optical adhesive, ergo 8500)	-	Solid	Two and three-photon imaging, laser speckle contrast imaging, hyperspectral imaging, brighfield imaging at 550nm, two-photon calcium imaging	Imaging depth, signal intensity, minimum resolvable vascular diameter, CNR, signal to background ratio (SBR)	Narrow (Bihemispheric windows evaluated in the acute period)	Up to 21 days	Yes
**LCCW** Zhang et al.^11^	S1, S2, primer, joint and sealing gels	-	Solid	Optical coherence tomography, two-photon imaging	Imaging depth, signal intensity, CNR and vascular morphology parameters such as vascular density, and branching index	Narrow	2 months	Yes

Cyanoacrylate and methacrylate-based agents (e.g., C&B Metabond, Superbond, and Orthojet) have been previously used in thinned and intact skull preparations to obtain transparency.^12^^,^^14^^,^^26^^,^^35^^,^^36^^,^^37^^,^^38^^,^^39^^,^^40^^,^^41^ Transparent windows utilizing C&B Metabond and other methacrylate-based compounds on intact skulls have remained transparent for up to 2 months and have been used for awake widefield functional imaging for up to 25 weeks.^12^^,^^33^ Widefield transparent windows using multilayer cyanoacrylate applications have been used for awake widefield calcium imaging and have remained transparent for up to 5 months.^27^ Cyanoacrylate application has also been coupled with NOA81, a UV-curable optical adhesive similar to NOA61 used in this study, for awake widefield imaging for up to 7 months.^29^^,^^31^ A cyanoacrylate-based method named “transparent skull (TS)” has also been shown to remain transparent for up to 2 months in mice older than 18 weeks old, but the size of the imaging area was limited; small parietal windows were evaluated.^10^ TS was used to investigate critical period-like plasticity, was accessible for injections through the skull, but provided transparency limited to blood vessel visualization and was used in anesthetized mice.^10^

In 2012, an *in vivo* skull optical clearing technique by a topical solution named SOCS (skull optical clearing solution) was introduced, which included various chemicals including laurinol, ethylenediaminetetraacetic acid (EDTA), dimethyl sulfoxide, sorbitol, alcohol, glucose, and weak alkaline elements.^34^ SOCS was only used for acute LSCI of cortical vessels and did not provide dendritic resolution.^34^ It did not affect cortical blood flow distribution *in vivo* on LSCI.^34^ The concentration of the key component, laurinol, determined the skull optical clearing power of SOCS.^34^

Later, a reversible EDTA-based clearing window named “skull optical clearing window (SOCW)” was used to observe dendritic spine plasticity in critical periods and the impact of laser ablation on dendrites and microglia.^9^ In younger mice (aged 15 to 20 days old), collagenase was used to clear the skull, which depolymerized collagen on the skull surface.^9^^,^^42^ In adult mice, SOCW consisted of skull thinning prior to EDTA application, followed by glycerol for index-matching, which proved impractical for water-based objectives as a plastic layer between glycerol and water must be placed, compromising the stability of the preparation.^8^^,^^9^ EDTA acts by binding to calcium and sequestering it, decalcifying the bone, in addition to homogenizing the proteins.^42^ EDTA does not affect the structure of collagen; thus, the structural integrity of the skull remains undisturbed.^42^ Imaging resolution with SOCW, though worse than conventional windows, was at synaptic-resolution at superficial cortical layers with two-photon microscopy. As it was not a permanent window and was reversed after imaging with PBS, the thinned skull had to be re-cleared prior to each imaging session. Therefore, SOCW was not suitable for repeated awake imaging and was only used in anesthetized mice. Thinning of the skull also introduced bone regrowth as an issue, limited the size of the window, and rendered it unsuitable for widefield imaging. The authors did not observe any microglia movement, change in cerebrovascular morphology, change in GFAP expression, or microglial morphology after SOCW, compared with an open-skull window. Dendritic spine images up to 2 days after surgery and images displaying the distribution of vessels and microglia up to 21 days were evaluated, all of which required reclearing prior to imaging.

Then, a novel method named urea-based skull optical clearing agents (USOCA) was introduced, consisting of 2 solutions applied on an intact skull sequentially.^7^ Solution 1 (S1) was an ethanol-urea solution, and Solution 2 (S2) was high-concentration sodium dodecyl benzene sulfonate (SDBS), a type of detergent.^7^ The hydroxyl groups of ethanol in S1 dissolve collagen by forming hydrogen bond bridges with its structure.^7^^,^^43^ Urea in S1 is thought to aid in transparency through hydration of the tissue, leading to tissue expansion, diluting the complex elements of the skull, in addition to making tissue more permeable to alcohols and detergents, and has been frequently used in *ex vivo* tissue clearing, mixed with detergent solutions in some methods.^44^^–^^50^ Detergents such as sodium dodecyl sulfate have been previously used in *ex vivo* tissue clearing of the brain through its lipid removal capability from tissue.^51^^,^^52^ SDBS in S2 also removes lipids.^8^^,^^42^ In summary, S1 depolymerizes collagen’s triple helix structure, whereas S2 removes lipids and homogenizes collagens, making USOCA a powerful protein dissolver.^8^^,^^42^ USOCA was used to observe hemodynamic changes after middle cerebral artery occlusion. It provided long-term imaging for up to 6 months in 2-month-old mice with repeated clearing, with better clearing efficacy than SOCW.^7^ USOCA was effective in 2- to 8-month-old mice, but had considerable limitations. Parameters of clearing efficacy were only measured under anesthesia, and bihemispheric wide-scale imaging parameters were only measured in the acute period for up to 30 min. USOCA was unfit for awake imaging because it required re-clearing with liquid agents before imaging, which had to be reapplied for extended imaging durations. Transparency of the skull also decreased with repeated USOCA application, limiting the number of imaging sessions.^8^ Safety of the window was tested, with *in vivo* monitoring of microglial activation 1 h after clearing and *in vitro* monitoring 2 days after clearing, in addition to GFAP staining 10 days after clearing. USOCA did not lead to microglial activation.^7^ Hematoxylin-eosin staining of the liver and the kidney of the animals along with organ-to-body ratios showed no signs of metabolic toxicity.^7^ Body weights of the animals did not show major changes after the procedure.^7^ In later studies, USOCA has been used to acutely monitor photodynamic therapy-induced blood-brain barrier permeability with spectral, confocal, and two-photon imaging in 4- and 8-week-old mice with a narrow window size.^53^^–^^55^ USOCA did not disrupt BBB integrity.^53^^–^^55^ USOCA has also been used for photothrombosis and imaging for up to 14 days.^56^ Bihemispheric LSCI images were assessed in the acute period, but only a small area has been evaluated chronically.^56^ Microglia were not activated on day 14 in response to repetitive clearing with USOCA.^56^ Body weights of mice also did not differ from the sham group.^56^

For chronic longitudinal awake imaging, USOCA was later combined with optical adhesives and gels to obtain a solid window ready for imaging.^8^^,^^11^ A solid window permits awake imaging and provides a ready-to-image window without any pre-imaging preparation. In one method called Through-Intact-Skull (TIS) window, a UV-curable optical adhesive (ergo 8500, Kisling, Switzerland) named S3 was combined with USOCA.^8^ S3 was used for refractive index matching in addition to solidifying the window and preserving transparency as with each re-clearing, the skull opacity gradually increases.^8^ TIS was tested for up to 21 days with two- and three-photon microscopy and LSCI to observe immune responses after traumatic brain injury. Transparency of the window measured by CNR decreased at 21 days compared with day 0 to 14. Bihemispheric images up to 4 h were displayed, and the long-term transparency of the bihemispheric window cannot be evaluated. TIS window provided higher resolution than SOCW and USOCA, which enabled imaging dendritic spines, and was ideal for use in mice under 3 months old.^8^ TIS also provided awake imaging at long durations because the window was solid and stable, and no reapplication of solutions was needed. The authors observed no position or shape changes of neurons or blood vessels long term, no activation or migration of microglia (1 h and 2 days after TIS *in vivo*, 2 and 21 days after clearing on brain slices), no astrocytic activation with GFAP staining 10 and 21 days after TIS, and no neutrophils or any inflammatory changes in the parenchyma on H&E staining 2 days after clearing and concluded that TIS did not induce inflammation. In another method, primer, joint, and sealing gels were combined with USOCA, named “long-term clearing cranial window (LCCW)” and used for photothrombosis in 8-10-week-old awake mice, as well as OCT and two-photon imaging to observe the recovery of cortical and calvarial blood vessels after ischemic injury.^11^ LCCW remained transparent for up to two months; however, only small fields of view were evaluated, thus the efficacy for widefield chronic use was not clear.^11^ LCCW did not change the density or orientation of blood vessels over the long term or induce migration of microglia in the cortex and monocytes in the skull bone marrow within 1 and 24 h of clearing, respectively, indicating no inflammatory changes or noticeable effects on the brain microenvironment.^11^

The mechanisms of clearing of the previously introduced methods (SOCW and USOCA) were explored in an acute setting in a later study, using three-photon microscopy and label-free hyperspectral stimulated Raman scattering microscopy.^42^ Only a small region of the skull was cleared in this study. USOCA was a more effective protein dissolver than collagenase.^42^ A modified clearing method involving the application of saline, USOCA, and SOCW sequentially was introduced and provided clear imaging of cortical vessels deeper than 850  μm with three-photon imaging, similar to TIS window (800 to 900  μm with three-photon imaging), compared with up to 250  μm with SOCW and around 300  μm with USOCA alone, both with two-photon imaging.^7^^–^^9^^,^^42^ By combining EDTA and USOCA, this method dissolved both the organic and inorganic parts of the skull, and the authors suggested the corrosivity of EDTA may decrease when combined with USOCA or glycerol.^42^ H&E staining of brain slices showed no microenvironmental changes with clearing.^42^

For long-term functional imaging of the cortical surface in awake mice, a ready-to-use bihemispheric imaging window is needed.^12^ In light of these results to date, we decided to systematically compare several methods of skull optical clearing for chronic mesoscopic imaging in awake mice. The first method is cyanoacrylate/methyl methacrylate-based, which has been shown to maintain long-term transparency in bihemispheric windows. Second, we tested a UV-cured adhesive (NOA-61) with or without a calcium-chelating agent (EDTA).^9^ Lastly, we combined USOCA with a UV-cured adhesive similar to the component “S3” used in the TIS window.^7^^,^^8^ Our overall aim is to provide a reference for investigators planning to utilize skull-clearing techniques for optical mesoscopic imaging with little prior expertise.

## 2 Materials and Methods

### 2.1 Animals

For imaging, 8 to 16-week-old C57BL/6 wild-type mice (n=34) and C57BL/6J-Thy1-COP4/EYFP mice (n=6) (The Jackson Laboratory, strain: 007612) were used. Mice were maintained at 12:12 light/dark cycle and a stable temperature (19°C to 22°C) with ad libitum access to food and water. All experiments and animal procedures were approved by the Hacettepe University Animal Experimentations Local Ethics Board (Approval number: 2022/40).

### 2.2 Surgical Procedure

Surgical instruments were sterilized in a hot bead sterilizer (Fine Science Tools, USA) for 20 s. Prior to surgery, cover glasses (0101242, 0.13 to 0.16 mm thick, Paul Marienfeld GmbH & Co. KG, Germany) were cut by a diamond pen to fit the final window size (10 mm diameter).

Mice were anesthetized with isoflurane (4% induction, 1% to 1.5% maintenance in oxygen) head-fixed in a stereotaxic frame (World Precision Instruments, Florida, United States) under a stereomicroscope (SMZ745T, Nikon Instruments Inc., Japan). Temperature was maintained at 37.0°C on a homeothermic blanket control unit (Kent Scientific, Torrington, United States) and monitored with a rectal probe. Hindlimb reflexes were checked periodically to ensure depth of anesthesia. Eye ointment (Recugel, Bausch + Lomb, Ontario, Canada) was copiously applied throughout surgery as needed to protect the cornea from keratitis.

Following hair removal with depilatory cream, the scalp was disinfected with 70% ethanol followed by 10% povidone-iodine (e.g., Betadine), and a second application of 70% ethanol from the eyeline to the neckline. A midline incision was made to the scalp, large enough to visualize both hemispheres (both temporalis muscles, frontal and parietal, and interparietal bones). The periosteum was gently removed with a cotton swab, and the skull was dried with a pressurized clean air spray. Both temporalis muscles were retracted from their insertion, avoiding damage to the superficial temporal vein,^57^ and the remaining groove was filled with cyanoacrylate glue (Loctite 401, Henkel, Germany) to provide a solid surface for head plate adhesion.

For better adhesion, crosshatch patterns were made over the area where the head plate will be glued with a #11 blade, with attention not to cause bleeding or injury to the skull. Skull was again dried with clean pressurized air spray before gluing the head plate. Prior to mounting the head plate, the skull surface must be completely dry and devoid of any soft tissue. Accidental head plate adhesion to soft tissue will lead to a nonstable attachment and detachments long-term, in addition to unwanted motion of region of interest during imaging.

To mount the head plate, a thin layer of cyanoacrylate glue was spread over the surface, and a cotton swab was swept over the inner rim to remove the excess glue. Then, the head plate was glued to the skull and gently pressed against the skull for 15 to 20 s. Care was taken not to apply excessive glue to prevent its spillage over to the region of interest. On the other hand, too little cyanoacrylate applied to the head plate was also avoided as it could lead to head plate detachments.

After optical clearing, the window was immediately closed with a custom-made cap to prevent glue spillage over the window in the following steps. Then, the outer rim of the head plate was covered with a layer of cyanoacrylate glue, a dental cement layer (Meliodent, Kulzer, Germany), and a second cyanoacrylate glue layer to prevent detachment of the head plate from the skull, covering all exposed skull around the window.

Eyes and whiskers were prevented from coming in contact with cyanoacrylate or dental cement as this could lead to blindness and clumping of the whiskers.

After surgery, the animal was returned to a clean cage, placed on a heating pad for recovery, and monitored hourly until fully recovered from anesthesia. Oral ibuprofen in the drinking water (50  mg/100  ml) was provided for postoperative analgesia for 3 days (1 day in interhemispheric homotopic connectivity experiments). Cages were arranged to contain minimal nesting material postsurgery as it could stick to the holders of the head plate, causing distress to the animal, or lead to dust build-up over the window.

### 2.3 Habituation to the Imaging Setup for Awake Head-Fixed Imaging

**Fig. 1:**
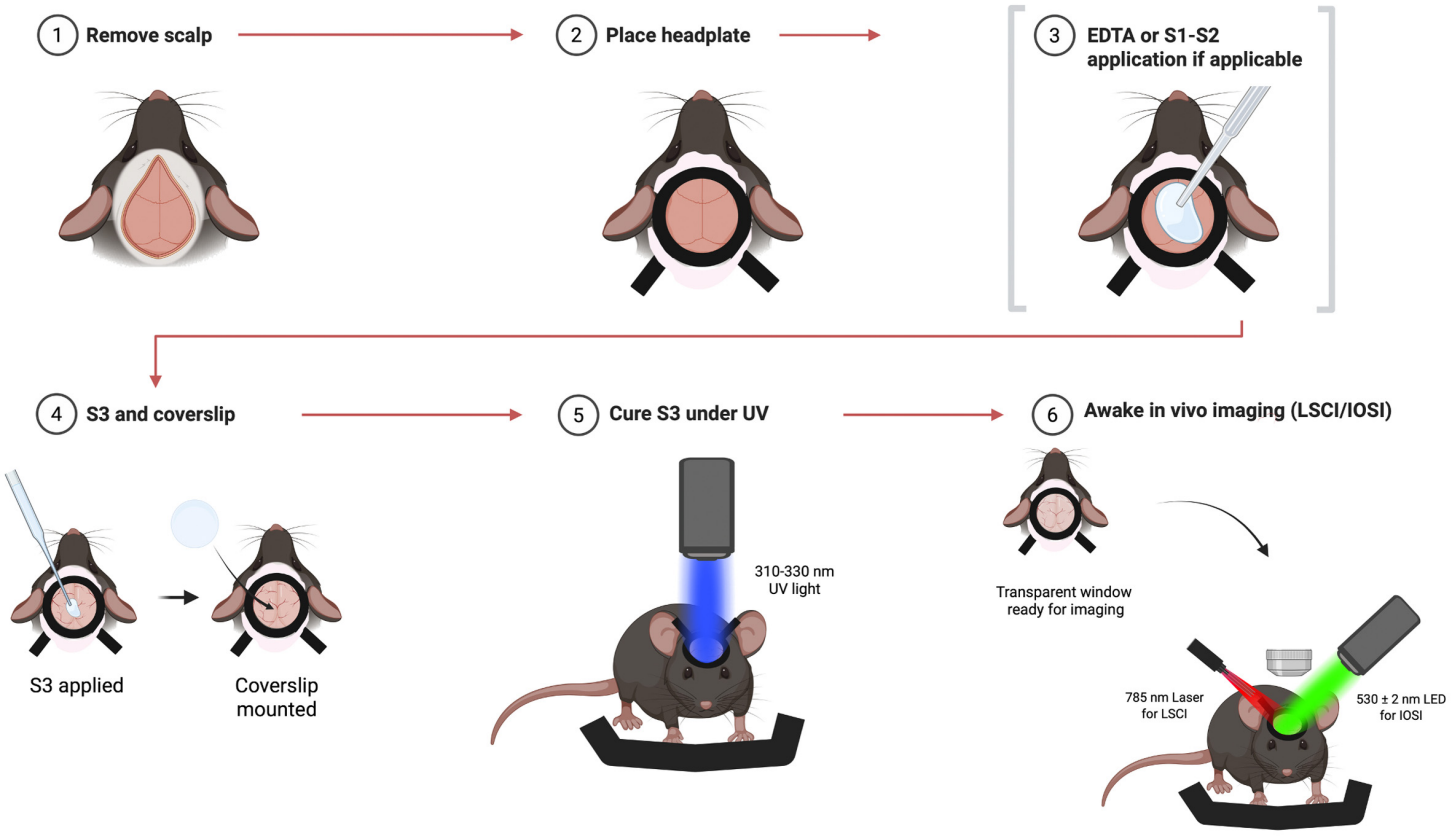
Surgery and skull optical clearing with UV-cured methods. NOA-61 method skips step 3, whereas EDTA-NOA61 method involves EDTA application in step 3, and Triple-S involves application of S1 followed by S2.

Following recovery, starting on the day of the procedure (Day 0), mice were habituated to the imaging setup, head-fixed in an imaging cradle, and attached to a holder from two attachment points on the posterior side of the head plate (the headplate can be visualized in Fig. 1). 3 sessions of habituation with durations of 5, 10, and 15 min were performed every day for 3 days, with the mice being rewarded every 5 min with sweetened condensed milk. For interhemispheric homotopic connectivity experiments, mice were habituated for 4 days (10, 20, 30, and 40 min a day) prior to imaging on day 5 after clearing. If the mice were restless or visibly stressed in the imaging cradle, the habituation session was terminated and the mouse was returned to its home cage.

During nonimaging periods, a custom-made 3D-printed cap interlocked to the head plate covered the transparent window to prevent damage to the window or dust buildup and to prevent light exposure of the Channelrhodopsin-2 positive transgenic animals.

### 2.4 Skull Optical Clearing

After the head plate is glued to the skull with cyanoacrylate, it is recommended to wait for up to 10 min before applying the clearing agents, to let the cyanoacrylate fully dry and prevent its mixture with clearing agents. Surgery and skull optical clearing steps can be seen in Fig. 1. Testing order was randomized: Triple-S, EDTA-NOA61, and cyanoacrylate groups were tested in a randomized order. Testing order of the Metabond group was randomized with the NOA61 group.

#### 2.4.1 EDTA-NOA61 application

The EDTA solution is 10% EDTA disodium (AppliChem, Darmstadt, Germany) in 1X PBS (Sigma-Aldrich, USA); pH is kept between 7.48 and 7.60. As 10% PBS-EDTA solution loses its clearing power with time, we recommend that it be made fresh every 2 to 3 days for ideal results. It can be stored in +4°C. EDTA was applied for 20 to 30 min.

To obtain a ready-to-use chronic transparent window and maintain a solid transparent window for long-term chronic imaging, we use a clear, colorless, UV-curable liquid photopolymer as our optical adhesive (NOA61, Norland Products, USA). NOA61 has a urethane-related resin-based formulation and consists of mercapto esters (50-65%) and triallyl isocyanurate (30% to 55%). It transmits visible light nearly 100% has high transmittance in the 400 to 3000 nm range, with a refractive index of 1.56 after curing, similar to the optical adhesive S3.^8^

Because NOA61 adheres to the internal rim of the head plate, leading to an uneven surface, we recommend that only a small amount covering the central part of the window be applied first, covered with a cover glass, after which NOA61 can be applied from the gaps between the outer rim of the head plate and the cover glass with a narrow instrument, with attention not to spill any drops on the cover glass. NOA61 easily forms bubbles, which must be carefully removed before placing the cover glass and prior to UV curing of NOA61. After curing, bubbles will become permanent and disrupt the quality of the imaging window. When the window is ready, it is then cured for 2 to 5 min under 310 to 330 nm UV light until NOA61 is fully solid. NOA61 can generate heat during curing, and while we did not observe thermal damage (Iba1-stained sections display no difference in the number of microglia compared with naive animals or spontaneously cured methods such as cyanoacrylate and Metabond), experimenters should be cautious of possible thermal damage with extended durations of curing.

#### 2.4.2 NOA61 application

For NOA61 application without EDTA, NOA61 was applied on the skull after head plate attachment and cured under UV light as described above.

#### 2.4.3 TripleS (3S) application

S1 is a 75% (v/v) ethanol (Isolab, Germany) and urea (Sigma-Aldrich, USA) solution with a volume-mass ratio of 10:3.^7^ S2 is a 0.7M NaOH (Merck, Germany) and dodecylbenzenesulfonic acid (DDBSA) (Sigma-Aldrich, USA) solution with a volume-mass ratio of 24:5, with pH kept between 7.2 and 8.^7^ S1 and S2 can be stored at room temperature and can be used for up to 6 months, although for ideal results, they should be remade monthly. In previous studies, S1 and S2 were applied for 10 to 20 min and 5 min, respectively.^7^^,^^8^^,^^11^ To reduce the opacification of the skull while S2 was removed and S3 was applied, we applied S1 for 20 min and S2 for 10 min. S2 should be carefully manipulated and removed as it readily foams and forms bubbles due to its DDBSA content.

#### 2.4.4 Cyanoacrylate application

A single thin layer of cyanoacrylate (∼50 to 70  μl) was applied to the skull and immediately covered with a coverglass before it dried, as suggested previously with adhesive-applied windows.^12^^,^^38^ Curing time was ∼5 to 10 min.

#### 2.4.5 Methyl methacrylate application

A thin layer of O-80 (Imicryl, Turkey), a methyl-methacrylate product equivalent to Metabond, was applied to the skull and immediately covered with a coverglass before it dried. Although cyanoacrylate preparations (such as Zap-a-Gap) may maintain clarity without a cover glass, Metabond tends to become opaque due to scratches. A cover glass layer on top helps maintain long-term transparency and provides scratch resistance.

### 2.5 Imaging

All images were obtained when the mice were fully awake in the imaging cradle, but to place the animals in the imaging cradle, brief anesthesia with 4% isoflurane was used. The cover glass over the transparent window was cleaned with isopropyl alcohol before imaging for better visualization.

**Fig. 2:**
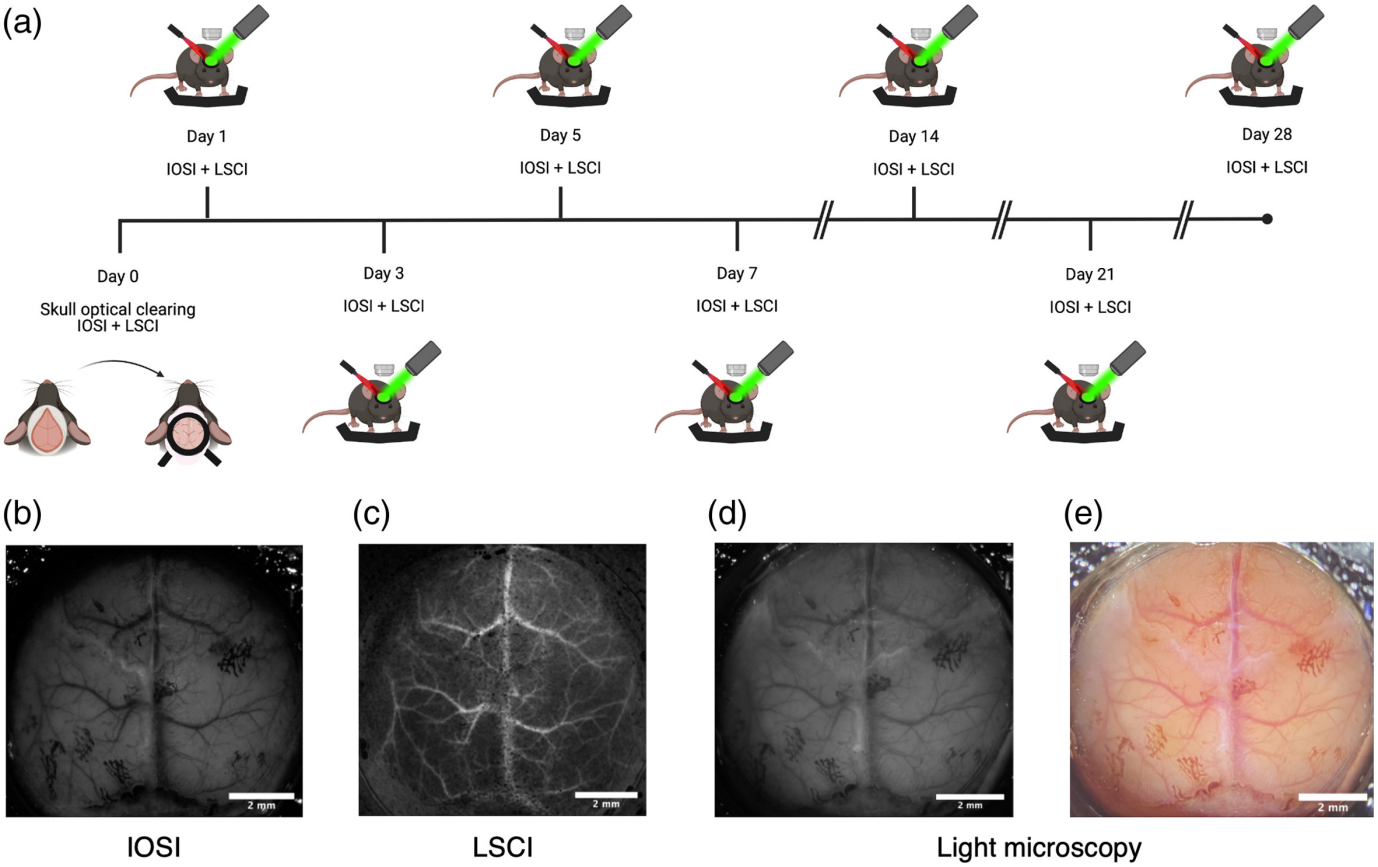
(a) Experimental timeline. (b)–(e) Representative images immediately after optical clearing with NOA61: (b) IOSI, (c) LSCI, and (d) and (e) light microscopy.

The experimental timeline can be seen in Fig. 2(a). LSCI and IOSI were performed on Day 0, Day 1, Day 3, Day 5, Day 7, Day 14, Day 21, and Day 28. During imaging, all animals were awake and head-fixed by their head plate with two attachment points to the imaging cradle for ideal stabilization.

A stereomicroscope (SMZ1000, Nikon Instruments Inc., Japan) mounted with a CCD camera (Basler acA1300-60gmNIR, Basler Vision Technologies, Germany) was used for IOSI and LSCI [see Fig. 2(a)]. Light microscope images were taken with another CCD camera (Nikon DS-Qi1Mc, Nikon Instruments Inc., Japan) using NIS Elements Version 3.22.15 (Nikon Instruments Inc., Japan). For IOSI, 530±2  nm LED light (Thorlabs, USA) was used to measure total hemoglobin concentration for IOS imaging, and 8-bit 1280×1024-pixel images were obtained. For interhemispheric homotopic connectivity analyses, 8-bit 512×512-pixel images were obtained through 2×2 binning of 1024×1024-pixel images. For LSCI, the window was illuminated using a 785-nm laser diode (Thorlabs, USA); 8-bit 1276×1020-pixel images were recorded.

### 2.6 Image Processing

For LSCI, every image set consisted of 15 raw speckle images, with an exposure time of 5 ms. After averaging the image sets, a spatial speckle contrast (K) image was computed using a sliding window of 7×7  pixels. Speckle contrast images were then converted to integrated correlation time (ICT) images ($1/K^{2}$) to obtain blood flow index values. LSCI image stacks were averaged (nine images per stack).

IOSI images were brain-masked (manually selecting a polygonal mask of only the pixels corresponding to the brain and excluding pixels corresponding to the outer head plate rim, also excluding any artifacts and/or inflammatory-like reaction areas during thresholding) and auto-thresholded using Otsu thresholding, after which the percentage of the area occupied by vasculature was calculated, used as a measure of transparency. A sample raw image and a brain-masked and auto-thresholded image pair can be seen in Fig. 4(a).

For the analysis of the area occluded by drying artifacts and/or bubbles, LSCI images were used. LSCI images were processed as mentioned above prior to analysis. Artifacts on LSCI images appear black and are easy to identify. A blinded researcher counted the occluded areas on one averaged LSCI image for each animal on Day 0 (day of the clearing procedure). Results were reported as % area occluded over the entire window, calculated by dividing total occluded area size by total window size. Sample images from cyanoacrylate, Metabond, and NOA61 groups can be seen in Fig. 4(g).

For interhemispheric homotopic connectivity analyses, CSD was triggered after IOS imaging at baseline for 10 min and the cortical surface was imaged continuously for 32.7 min (3 fps). The first 500 frames, corresponding to ∼2.8  min of data after CSD induction in which the CSD wave was propagating over the surface of the hemisphere, were disregarded, and the remaining 30 min were analyzed as three 10-min blocks. An open-access MATLAB script was used to pre-process IOSI images.^58^ 512×512  pixel images were downsampled to 128×128  pixels, temporally detrended, bandpass-filtered (0.035 to 0.08 Hz), and globally regressed. Interhemispheric homotopic connectivity values were calculated by taking the correlation coefficient of a pixel with its symmetrical pixel in the contralateral hemisphere, mirrored over a line orthogonal to the midline determined by bregma and lambda coordinates. All pixel values were then Fisher z-transformed and averaged prior to statistical testing, reported as the “Interhemispheric Homotopic Connectivity Index.”

### 2.7 Induction of CSDs

CSD was triggered optogenetically in 8- to 10-week-old C57BL/6J-Thy1-COP4/EYFP mice, expressing light-activated Channelrhodopsin-2 in excitatory neurons. A fiberoptic cable (diameter: 200  μm, numerical aperture: 0.53, Doric Lenses, Canada) was placed on the right frontal bone in a near 60 deg angle to the skull over the transparent window. The skull was constantly illuminated by a 785 nm laser for simultaneous LSCI. For excitation, a 450-nm laser light source (Doric Lenses, Canada) was used, coupled to the fiber optic cable. Optical power at the illumination spot was 6 mW. CSD wave was triggered with a 10 s stimulation and visualized and confirmed with LSCI, taking advantage of the typical blood flow changes.^59^

### 2.8 Immunofluorescence Staining, Quantification and Morphological Analysis of Iba1(+) Microglia

Eight to 24-week-old mice were used (n=18). Four weeks after clearing was performed, mice were transcardially perfused with 0.04% heparinized saline and 4% paraformaldehyde (PFA) under deep anesthesia. Brains were post-fixed in 4% PFA and then cryoprotected in 30% sucrose at 4°C until they sank. 40-micron-thick coronal sections corresponding to the primary sensory area (anteroposterior coordinates^60^ relative to bregma were ∼−0.32±0.1) were obtained with a freezing cryostat (CT520, Dakewe, China). Free-floating sections were blocked with 10% normal goat serum. Sections were incubated with primary rabbit antibodies against Iba1 (1:200, FUJIFILM Wako Pure Chemical Corporation, Japan) at +4°C for 48 h, followed by secondary antibodies of goat anti-rabbit Cy3 (1:200, Jackson Immunoresearch, USA) at room temperature for 1 h. The sections were mounted in glycerol/PBS (1:1) medium containing 12.5  mg/ml sodium azide and 1  μl/ml Hoechst-33258 (H3569, Thermo Fisher Scientific, USA). Sections were then examined under a laser scanning confocal microscope (SP8, Leica GmbH, Germany) with a water immersion objective (25 ×, NA:0.95). Iba1-positive microglia were imaged in Z-stacks (512×512  pixels with a field of view of ∼443×443  μm) and counted and averaged in maximum projection images obtained from three sections, 40  μm apart. For Sholl analysis of microglia, 2048×2048  pixel Z-stacks were imaged with 2.5 × zoom, yielding a field of view of ∼177×177  μm. Maximum projection images from Z-stacks were smoothed (Gaussian blur, σ=0.5), background was subtracted using a rolling ball radius of 50 pixels, and images were then auto-thresholded for binarization using the Otsu algorithm. Noise reduction was performed [remove outliers (radius = 2 pixels, threshold = 50) and analyze particles (minimum particle size = 10 pixels) algorithms], and a median filter (radius = 5 pixels) was applied prior to Sholl analysis. Sholl analysis was performed using Sholl Analysis from the simple neurite tracer (SNT) plugin on FIJI.^61^^,^^62^ 5-micrometer circles were drawn around the center of the soma of each microglial cell. Number of intersections at each radius was plotted. Identical parameters were applied to all images. All analyses were performed by researchers blinded to experimental groups.

### 2.9 Statistical Analyses

All images except for interhemispheric homotopic connectivity data were analyzed by FIJI 2.14 and 2.16^63^ and ImageJ 1.53a^64^ (National Institutes of Health, Bethesda, Maryland, USA). Statistical analyses were performed using GraphPad Prism Version 10.3.1 (GraphPad Software, Boston, Massachusetts, USA). Data were plotted as means ± SD. Normality of distribution was determined by the Shapiro–Wilk test. Groups were compared using one-way ANOVA, one-way ANOVA for repeated measures, Kruskal–Wallis, and Friedman tests. Tukey, Dunnett’s, and Dunn’s tests were used for multiple comparisons.

## 3 Results

### 3.1 Comparison of the Optical Clearing Performances of Three Different Methods for Longitudinal Mesoscopic Imaging

**Fig. 3:**
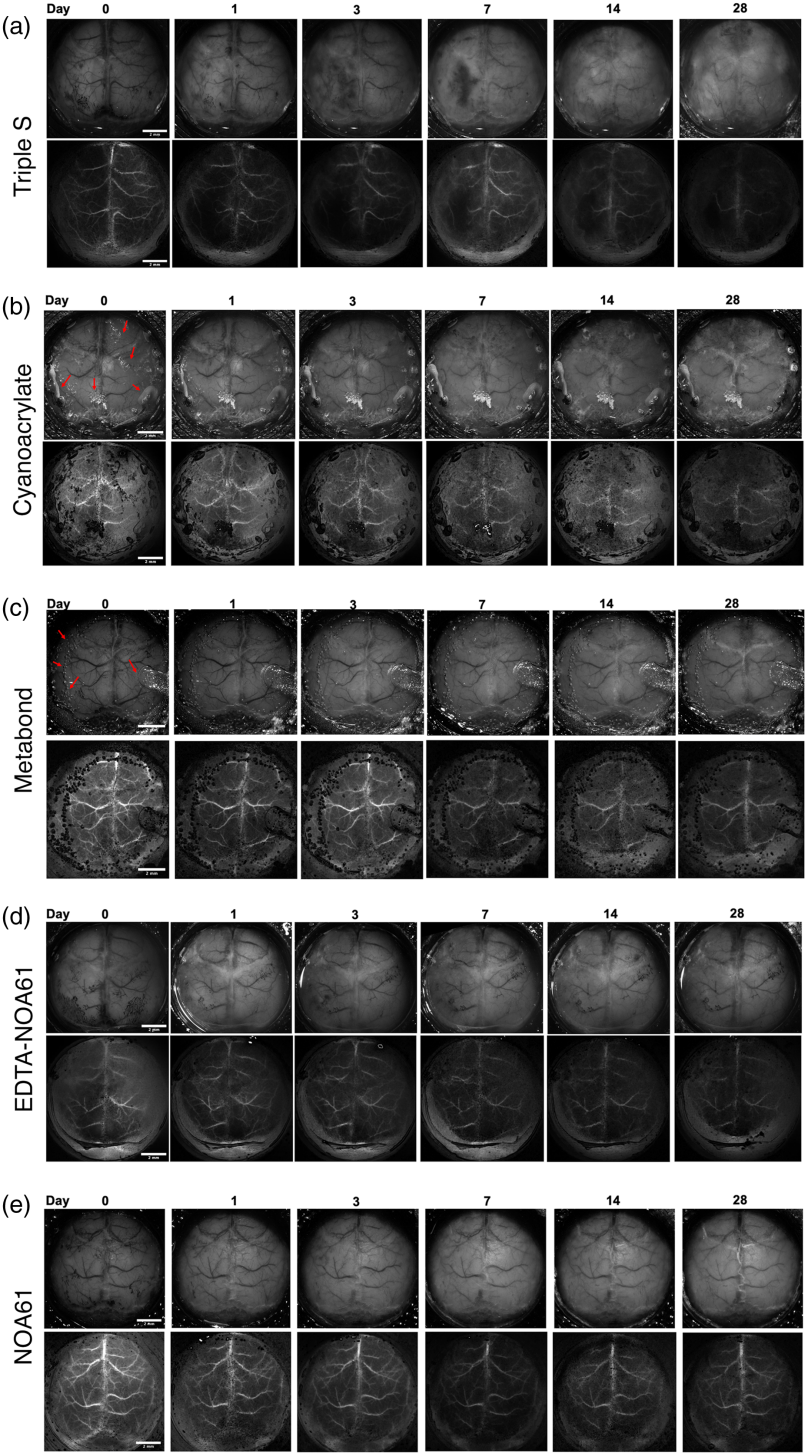
Longitudinal IOSI (top row) and LSCI (bottom row) images of (a) Triple-S, (b) Cyanocrylate, (c) Metabond, (d) EDTA-NOA61, and (e) NOA61-applied windows. Red arrows in panels (b) and (c) indicate artifacts in cyanoacrylate and Metabond methods. Scale bar = 2 mm.

**Fig. 4:**
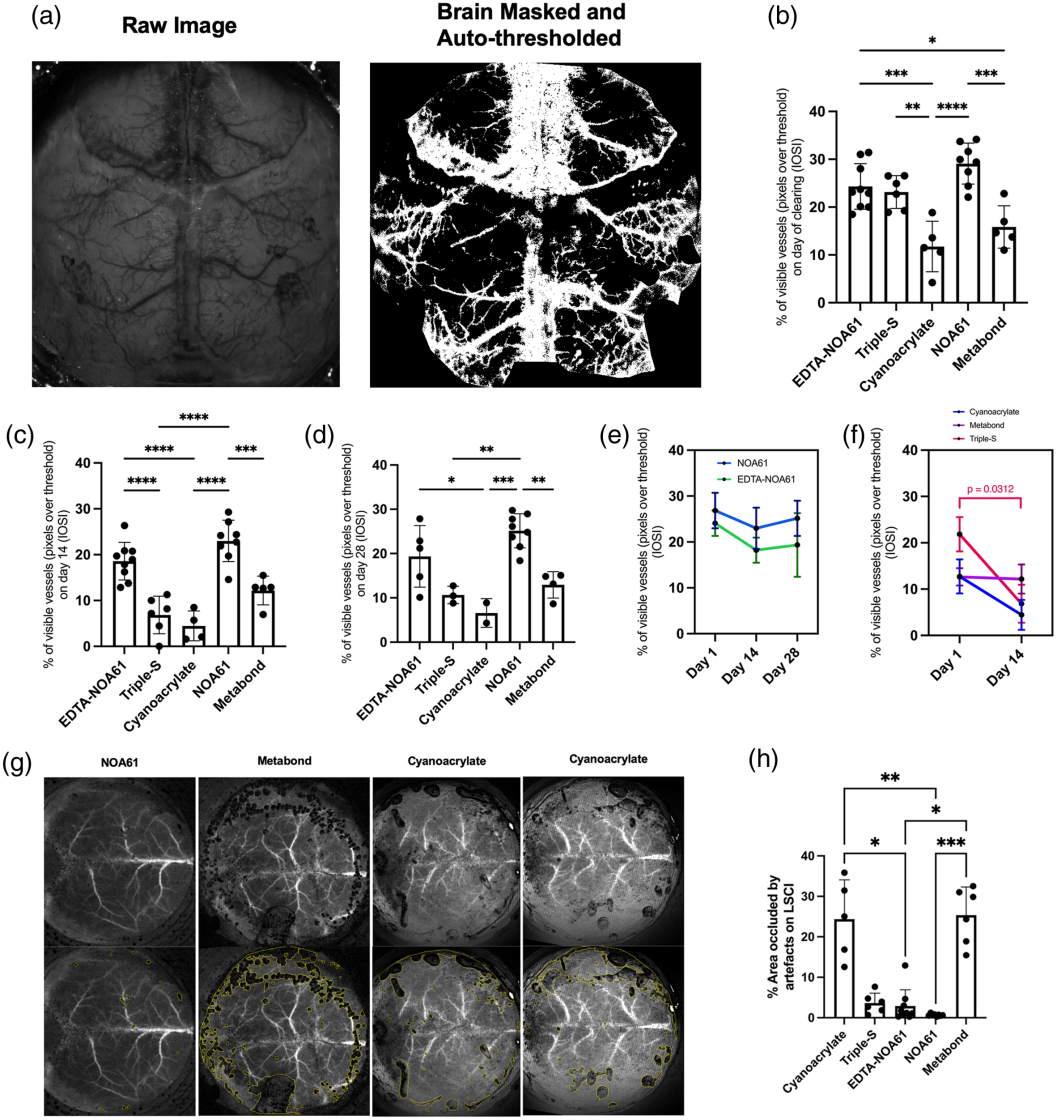
(a) Sample raw and brain-masked and auto-threshold (Otsu algorithm) images. (b)–(d) Percentage of auto-detected vasculature pixels (% of all image fields) with IOSI on Day 0 (day of clearing), Day 14 and Day 28 for all five methods of clearing. One-way ANOVA test, Tukey test for multiple comparisons. (e) Change in the percentage of auto-detected vasculature pixels (% of all image fields) with IOSI over 14 or 28 days for all five methods, with reference to Day 1. Data from Day 1-28 (NOA61, EDTA-NOA61 groups) and Day 1-14 (Cyanoacrylate, Metabond, Triple-S groups) was compared using the Friedman test (NOA61, EDTA-NOA61 groups), or Wilcoxon test (Cyanoacrylate, Metabond, and Triple-S groups). For NOA61 EDTA-NOA61 groups, pairwise comparisons between each timepoint and Day 1 were conducted using Dunn’s test. (f) Sample LSCI (Day 0) images (left panel) and images with areas occluded by artifacts/bubbles lineated in yellow (right panel) for cyanoacrylate, Metabond, and NOA61 methods. (g) Percentage of areas over the transparent window occluded by artifacts for all methods. Vertical bars indicate mean ± standard deviation. *P<0.05, **P<0.01, ***P<0.001, ****P<0.0001.

Acute application of the Triple-S technique, i.e., S1 (urea and ethanol), S2 (DDBSA and NaOH), and an optical clearing adhesive (NOA61), yielded a high-quality transparent window that can allow high-resolution widefield imaging with homogenous optical clearing, which was stable initially [Figs. 3(a), 4(b)–4(d), 4(f)]. However, starting around days 3 to 7 and becoming more apparent between days 7 to 21, progressive inflammatory-like reactions made the whole window unimageable with near-total opacification in three out of six mice tested. Fig. 4(b) in the Supplementary Material shows sample animals that showed total opacification. In two mice, we observed deterioration in parts of the window; sample mice from this group can be seen in Fig. 3(a) and Fig. 4(a) in the Supplementary Material. One mouse out of 6 mice showed no signs of an inflammatory-like reaction [Fig. 4(c) in the Supplementary Material]. In light of these results, we found Triple-S does not provide a consistent global transparency, deteriorating over time in a significant fraction of animals.

Cyanoacrylate application led to a heterogenous result, with certain areas showing good-quality clearing. Window quality remained relatively stable over 4 weeks, showing a slight but nonsignificant decline [Figs. 3(b) and 4]. However, in adjacent areas, most prominent near the edges of the window, drying artifacts of cyanoacrylate appeared 10 to 20 min after its application and did not improve over time (Fig. 1 in the Supplementary Material). Although this may depend on the surgeon’s performance in applying cyanoacrylate in as smooth manner as possible, this feature of a cyanoacrylate-only method was previously reported^38^ and thus should be considered as a factor for standardized studies to be performed across different institutions as the drying of the glue makes the performance of this technique especially unpredictable. This heterogeneous form of clearing leading to loss of data at certain points was not considered suitable for mesoscopic imaging, particularly for longitudinal functional connectivity studies to be performed chronically in awake animals. All animals tested (n=5) resulted in this heterogeneous appearance. Performance of methyl-methacrylate (O-80, Imicryl, Turkey, a methyl-methacrylate product equivalent to Metabond) was similar to cyanoacrylate, also resulting in drying artifacts that occluded parts of the window in all tested animals (n=6).

To better elucidate the effect of prior surgical training on clearing performance, we compared the quality of the window on the day of clearing for all animals for each of the methods in sequential order of surgery (Fig. 3 in the Supplementary Material). We observed that while the quality of the window slightly fluctuates from animal to animal, it does not improve over time for any of the methods, decreasing the likelihood of surgeon’s training contribution to clearing performance.

UV-cured optical adhesives, such as NOA61, have also been used as single-agent methods to obtain optical transparency. NOA61 monotreatment led to superior transparency compared to cyanoacrylate/methylmethacrylate on the day of clearing (Day 0) and displayed superior transparency on Day 14 compared to both cyanoacrylate/methylmethacrylate and Triple S methods. Transparency with NOA61 monotreatment lasted for up to 28 days without major opacification/inflammatory-like reactions over the window [Fig. 3(e)]. We then decided to combine the previously described EDTA-based clearing^9^ with NOA61, which we abbreviate here as EDTA-NOA61. EDTA was previously shown to provide sufficient optical access^9^ but required the solution to be present on the skull, which was not applicable for awake animal applications in chronic setting. EDTA-NOA61 resulted in an acute transparency (measured as % of visible vessels) not different from 3S and better than cyanoacrylate [Fig. 3(b)], provided homogenous clearing suitable for mesoscopic imaging, allowing access to cortical vasculature bilaterally, and most importantly, remained stable for at least 2 weeks after its application, and though it started to degrade after that timepoint, it still allowed optical access at 1 month [Figs. 4(c)–4(d), 3(d)] (n=9 mice). Other than general mild opacification between 2 and 4 weeks, we did not detect any visible deterioration of the window’s imaging performance, by contrast with Triple-S. We did not detect any significant difference between EDTA-NOA61 and NOA61 monotreatment for vasculature visibility.

### 3.2 Microglial Staining Reveals No Evidence of an Inflammatory Response With Topical Skull Optical Clearing

**Fig. 5:**
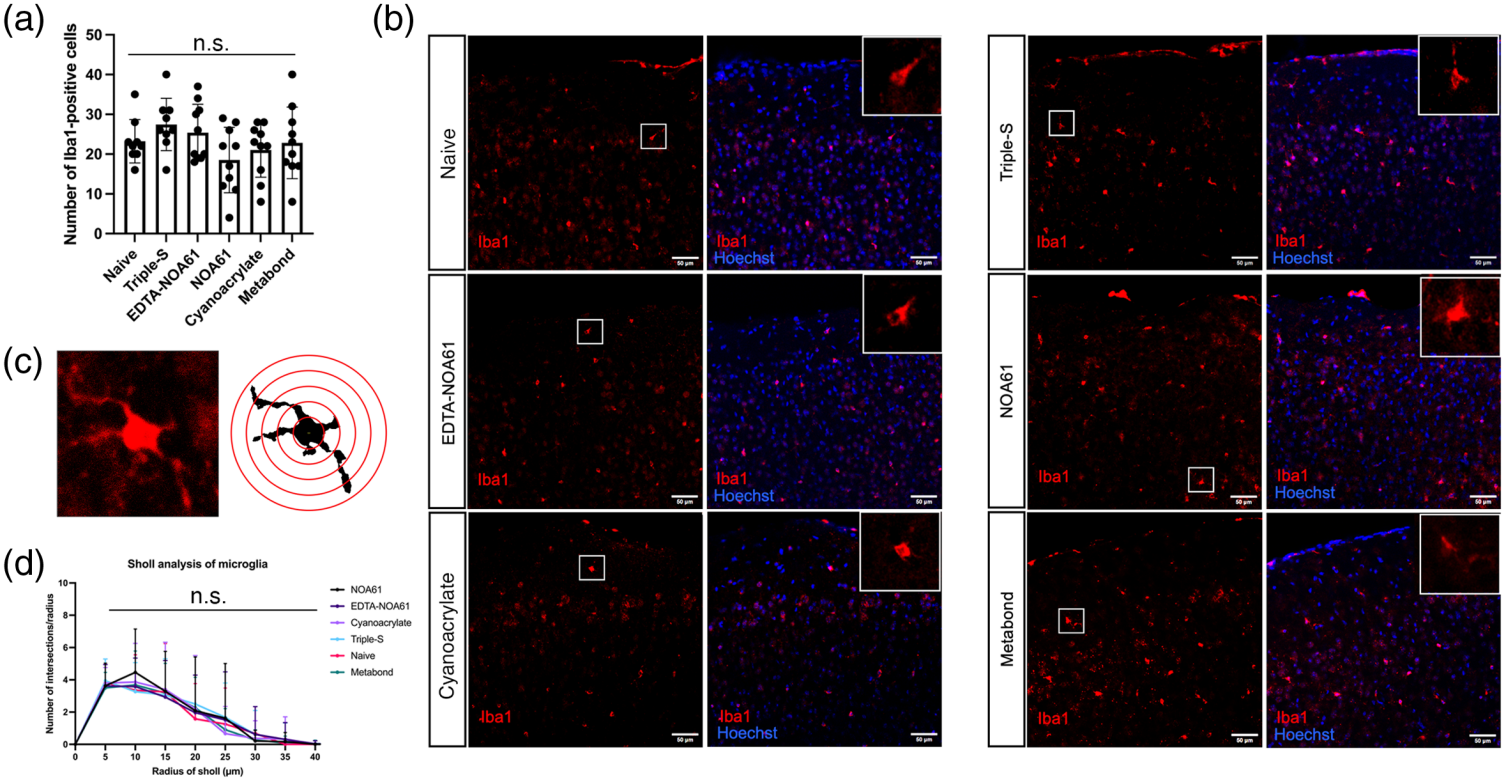
(a) Total number of microglia in a $443\times 443\;{\mu m}^{2}$ field of view from 5 skull optical clearing methods. One-way ANOVA test, Tukey test for multiple comparisons. (b) Representative Iba1-stained images from each method. Inset: Representative Iba1-positive cells. Scale bar=50  μm. (c) Representative microglial cell (raw image on the left, image processed and analyzed using Sholl analysis on the right). (d) Sholl analysis of microglia. Kruskal-Wallis test, Dunn’s test for multiple comparisons.

As the Triple-S technique led to opacification and areas resembling inflammation over the window, next, we investigated the possible inflammatory-like effects of Triple-S compared to other skull optical clearing methods. We observed no significant differences in the total number of microglia or complexity of microglial processes (Sholl analysis) between groups (Fig. 5). These results suggest that while Triple-S leads to an inflammatory-like reaction over the skull, which hinders brain imaging, this does not translate to an inflammatory effect in the superficial layers of the brain. We also highlight that there was no difference in the number of total Iba1-positive microglia in any of the topical skull optical clearing methods compared to the naive group, which supports topical skull optical clearing as a minimally invasive and safe procedure.

### 3.3 Application of a Candidate Skull Optical Technique for Functional Connectivity Changes after Optogenetically-Triggered CSDs

**Fig. 6:**
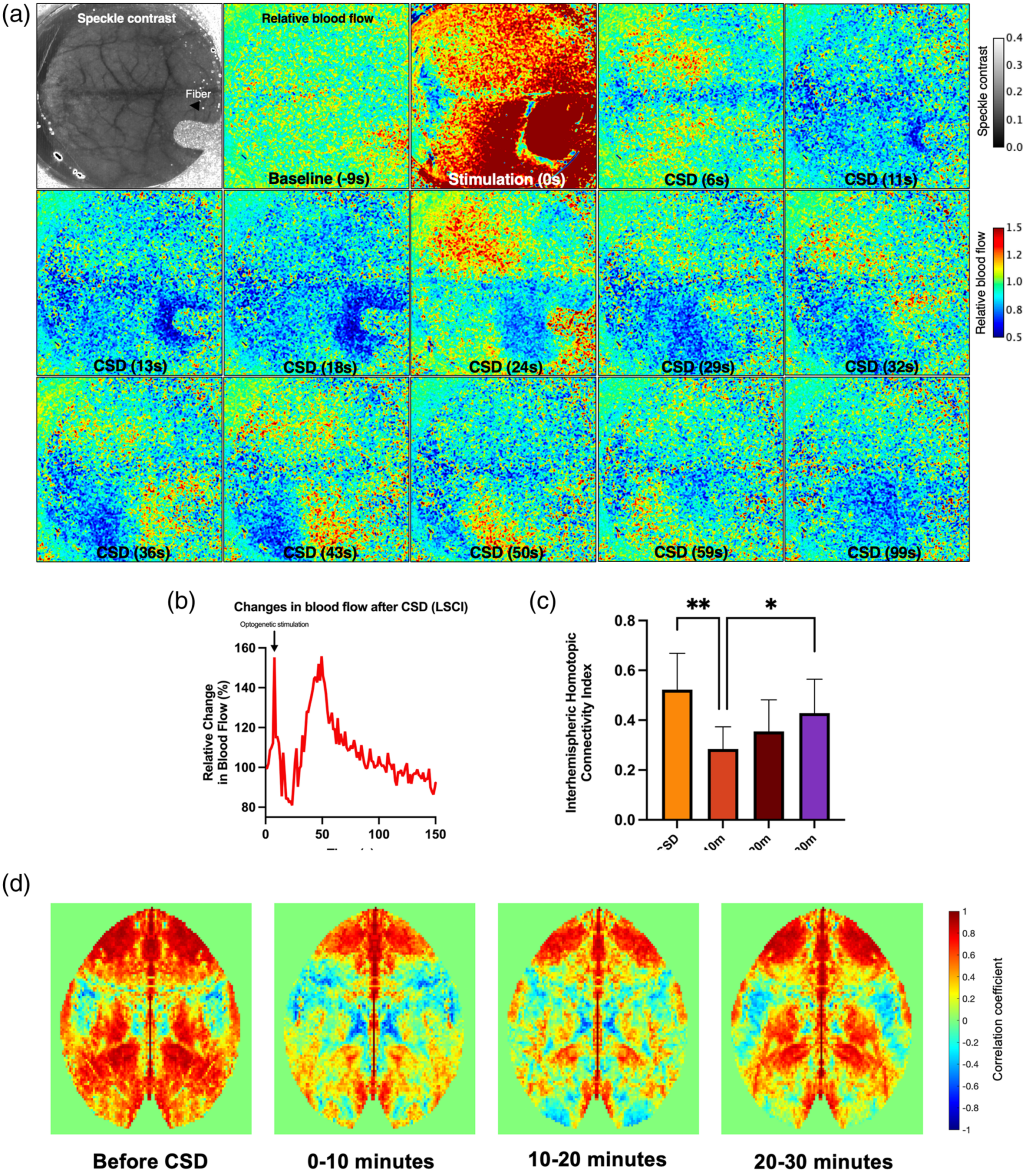
(a) Speckle contrast and relative blood flow maps as the CSD wave propagates unilaterally over the hemisphere. (b) Representative trace of relative blood flow changes in the ipsilateral cortex after CSD (c) Transient loss of ipsilateral functional connectivity following CSD (Fisher z-transformed). Vertical bars indicate mean ± standard deviation. *P<0.05, **P<0.01, Friedman test, Dunn’s multiple comparisons test. (d) Interhemispheric homotopic connectivity map changes in a representative animal before and after CSD. Color scale bars indicate Pearson correlation coefficients. Higher positive correlation coefficients are shown in red; higher negative correlation coefficients are shown in blue.

Based on our evaluation above, both NOA61 monotreatment and EDTA-NOA61 were found successful for providing consistent global optical access to the cortex chronically with a ready-to-image window for at least 1 month. We decided to utilize EDTA-NOA61 for imaging interhemispheric functional connectivity changes after optogenetically triggered CSD in awake mice. Chelation of calcium by EDTA, while not providing measurable addition to vasculature visibility, could improve the efficiency of light delivery to the cortex as calcium crystals in bone are a pronounced scattering element for visible light.^65^^,^^66^ CSD, the underlying mechanism of migraine aura, is a slow self-propagating wave of depolarization followed by depression of cortical activity, an established model of migraine aura in rodent studies.^67^ We aimed to test the immediate effects of the CSD wave on mesoscale functional connectivity in awake animals to demonstrate and test the use of a proposed well-performing skull optical clearing method for one of the various possible applications of mesoscopic skull optical clearing. We tested the EDTA-NOA61 method in Thy1-ChR2 transgenic mice expressing optogenetically activated ChR2 in excitatory neurons. After EDTA-NOA61 application, the animals were allowed to recover for 1 day and then were habituated for awake head-fixed optical imaging in the subsequent 4 days. After a baseline IOSI recording 5 days after initial EDTA-NOA61 application, we illuminated an ∼3-mm diameter spot using a 450-mm fiber coupled to a blue laser (6 mW), positioned at an ∼60  deg angle, in contact with the coverglass. CSD was triggered by a 10-s stimulation and confirmed by simultaneous LSCI^68^ [Figs. 6(a) and 6(b)]. Replicating the previously reported findings from awake rats with implanted guide cannulae and electrodes on the skull,^69^^,^^70^ we were able to show the transient loss of ipsilateral functional connectivity in our transgenic mice with minimally invasive transcranial approaches [Figs. 6(c)–6(d)].

## 4 Discussion

In this work, we compare existing *in vivo* optical skull-clearing methods in terms of their suitability for chronic widefield imaging in awake mice. EDTA-NOA61, which we found suitable for long-term widefield imaging in awake mice, was used to trigger CSD without craniotomy, measure chronic optical signals, and map functional connectivity through an intact skull. Avoiding the disadvantages of open-skull windows, it provided visibility of large cortical areas immediately after surgery and for up to 4 weeks without overt visible inflammatory changes. Compared with conventional thinned-skull windows, it provides a ready-to-image window, wide-scale optical access, and repeated imaging sessions over time without leading to bone regrowth.

The mechanism by which the Triple-S method creates an inflammatory-like reaction is unknown, but we hypothesize that the inflammatory-like reaction from S1-S2-S3 is due to the ethanol and urea content in S1 and the DDBSA content in S2. We have investigated whether S2 after EDTA (which slightly increases image quality) would lead to an inflammatory-like reaction, and shown S2 also leads to deterioration of the window by itself (Fig. 2 in the Supplementary Material). How quickly the inflammatory-like reaction progresses after Triple-S may differ between mice, and though in general deterioration of window quality becomes prominent around 7 to 21 days, it is most probably already in progress in earlier days, affecting captured data in imaging studies and making the use of the 3S method impractical. EDTA might be non-inflammatory compared to S1 and S2 because it does not act on collagen, the organic part of the skull and rather acts only on hydroxyapatite crystals, the inorganic part of the skull.^42^

Cyanoacrylate by itself without polishing agents is challenging to apply evenly and, in our experience, requires high surgical proficiency for wide-field use. Especially in widefield imaging studies where every pixel counts and carries valuable information for computation, cyanoacrylate and Metabond pose a potential risk for losing access to a population of pixels, compromising the quality of data. Computation of interhemispheric homotopic connectivity used in this study for instance, requires high-quality signals from every pixel on both hemispheres for reliable results. Reliability of the chosen clearing method is also important for animal welfare efforts, as it may help reduce the number of animals required per study, complying with the reduction principle of the 3Rs of animal research.^71^ It was previously reported that cyanoacrylate indeed led to these artifacts, even the Metabond-only method recommended by the authors in place of cyanoacrylate led to bubbles and drying artifacts over the window, significantly occluding certain regions of interest in widefield functional imaging.^38^^,^^39^ Our observations with a Metabond-equivalent method also led to bubbles and drying artifacts. Most cyanoacrylate and other self-cured adhesive-based applications in the literature make use of various modifications (including additional clearing and polishing agents and/or adjustments to make cyanoacrylate easier-to-use and more homogenous).^10^^,^^26^^,^^27^^,^^36^^,^^40^ For example, a cyanoacrylate-only method has been shown to remain transparent for up to 5 months, with minimal drying artifacts compared with our cyanoacrylate preparation, where the authors recommend applying it in multiple layers, starting at the sutures and progressing to the edges of the window with outer cement walls.^27^^,^^28^ Another method named “clear-skull cap” used cyanoacrylate, followed by a thin layer of clear dental acrylic, polishing with acrypoints, and a thin layer of clear nail polish, with imaging after 1 to 8 weeks of clearing.^26^ In this study, we aimed to provide guidance for choosing a skull optical clearing method for a surgeon unfamiliar with skull optical clearing. In our experience, self-cured cyanoacrylate- and methyl-methacrylate-based methods, compared with UV-cured NOA61-based methods, are especially challenging as they have minimal margin for error and are applied in a time-constrained setting because window quality cannot be improved once the cover glass is placed. Of note, a portion of the artifacts only develop after cyanoacrylate cures, at which point it is impossible to correct these to improve window quality. UV-curable methods, on the other hand, display low susceptibility to operator and provide sufficient time to perfect the quality of the window, after which the surgeon can decide to cure the window permanently. We therefore conclude that UV-curable methods are less challenging to apply and lead to less artifacts occluding the window compared to spontaneously cured methods.

EDTA-NOA61 combines chemicals formerly used in skull optical clearing^8^^,^^9^ and produces comparable results. EDTA was previously used as an *in vivo* skull optical clearing agent; however, the described clearing method was not suitable for repetitive awake imaging and did not involve wide-field application. Building on the previously described EDTA-based clearing method,^9^ we added a UV-curable optical adhesive to maintain the skull transparency chronically; a similar adhesive was previously combined with USOCA.^8^ In this study, we assessed the efficacy of clearing techniques in 8 to 16 week-old mice. As mice age, the increase in skull thickness may lead to suboptimal clearing results with topical agents.^8^^,^^9^

NOA61 by itself also produces comparable results to EDTA-NOA61 for optical access for functional imaging of the cortical surface. NOA81, a UV-curable adhesive similar to NOA81, has been used with cyanoacrylate in previous studies for long-term widefield imaging for up to 7 months.^29^^,^^31^ EDTA application before NOA61 may be a valuable addition for acoustic transparency for ultrasound access in addition to optical access. EDTA has been recently used to achieve acoustically transparent windows and better image quality in functional ultrasound imaging in rodents for up to 2 weeks and acoustic transparency in ex vivo human skulls.^72^ We conclude that to obtain optical-only access for widefield optical imaging, NOA61 is sufficient, whereas if the experimenter also utilizes concomitant functional ultrasound, EDTA-NOA61 can provide acoustic transparency in addition to optical transparency. NOA61 preparation provides easy surgery, quick recovery, instant imaging, and chronic long-term awake imaging. It is a minimally invasive, low-cost method does not require maintenance or repeated clearing, which are clear advantages. It provides stable transparency, allows awake imaging on a mesoscopic scale without inducing an inflammatory-like reaction, and is therefore safe and effective for longitudinal functional imaging. One of the advantages of NOA61 over SOCW is that because there is no need for thinning in adult mice, it does not come with the disadvantages of a thinned-skull preparation that could still trigger inflammatory changes^15^^,^^20^^,^^73^ yet produces comparable long-term results. Moreover, as a solid window, NOA61 also spares the repetitive exposure of EDTA or 3S to the skull, preserving the normal state of the brain. Experimenters may tailor these methods and choose a clearing method best suited to their experimental needs. Further research is needed to investigate the suitability and yield of NOA61 for other imaging techniques such as optical coherence tomography and two-photon imaging and for optogenetic modulation in other models.

## 5 Conclusion

Here, we evaluated different techniques for skull optical clearing for long-term surface-level cerebrovascular imaging of awake mice. Although different clearing methods may display unique advantages and disadvantages for different imaging approaches and experimental needs, we observed that NOA61-based skull optical clearing provides minimally invasive long-term monitoring of cerebrovascular hemodynamics, allowing us to image the near-normal physiological state of the brain *in vivo* without confounding effects of such anesthesia and inflammation, and is therefore a safe and viable method for awake chronic through-bone wide-field imaging.

## Supplementary Material

10.1117/1.NPh.13.2.025006.s01

## Data Availability

The data sets generated and analyzed during the current study are available on FigShare repository (https://doi.org/10.6084/m9.figshare.c.7933739). For functional connectivity analysis, publicly available MATLAB code by Boston University Neurophotonics Center was used (https://github.com/BUNPC/rsfcIOS).
