# Supplementary material for: Choosing a skull clearing technique for chronic mesoscopic optical imaging in awake mice
Source: Neurophotonics. 2026 Mar 24;13(2):025006. doi: 10.1117/1.NPh.13.2.025006 (PMC13012183; doi:10.1117/1.NPh.13.2.025006)
Supplement: Supplementary file 1 [file NPh_013_025006_SD001.pdf]

Supplementary Figures

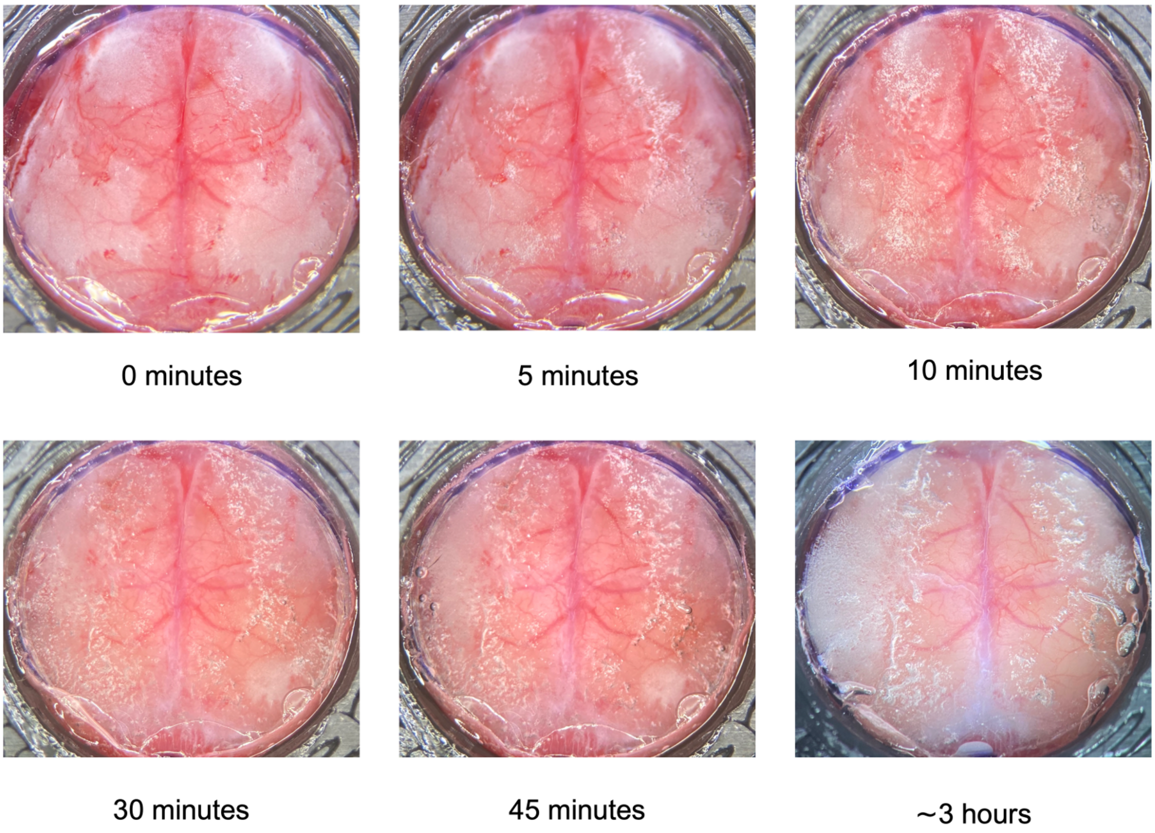

Figure S1 Drying of cyanoacrylate glue leads to unpredictable drying artifacts

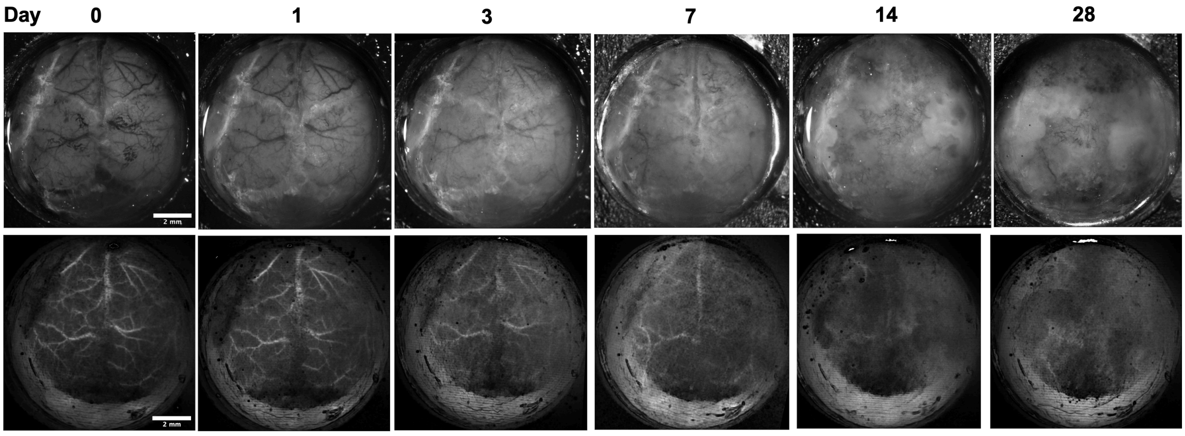

Figure S2 IOSI (top row) and LSCI (bottom row) images of a EDTA-S2-S3 applied window for 3 weeks.

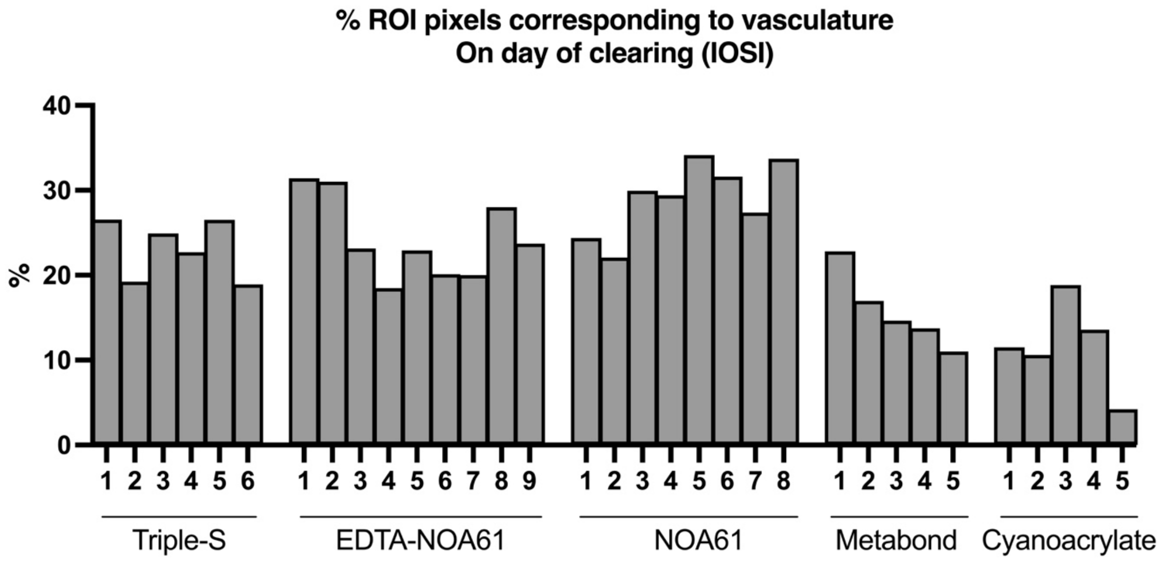

Figure S3 Percentage of auto-detected vasculature pixels (% of all image field) with IOSI on Day 0 (day of clearing) in order of clearing of animals for each method.

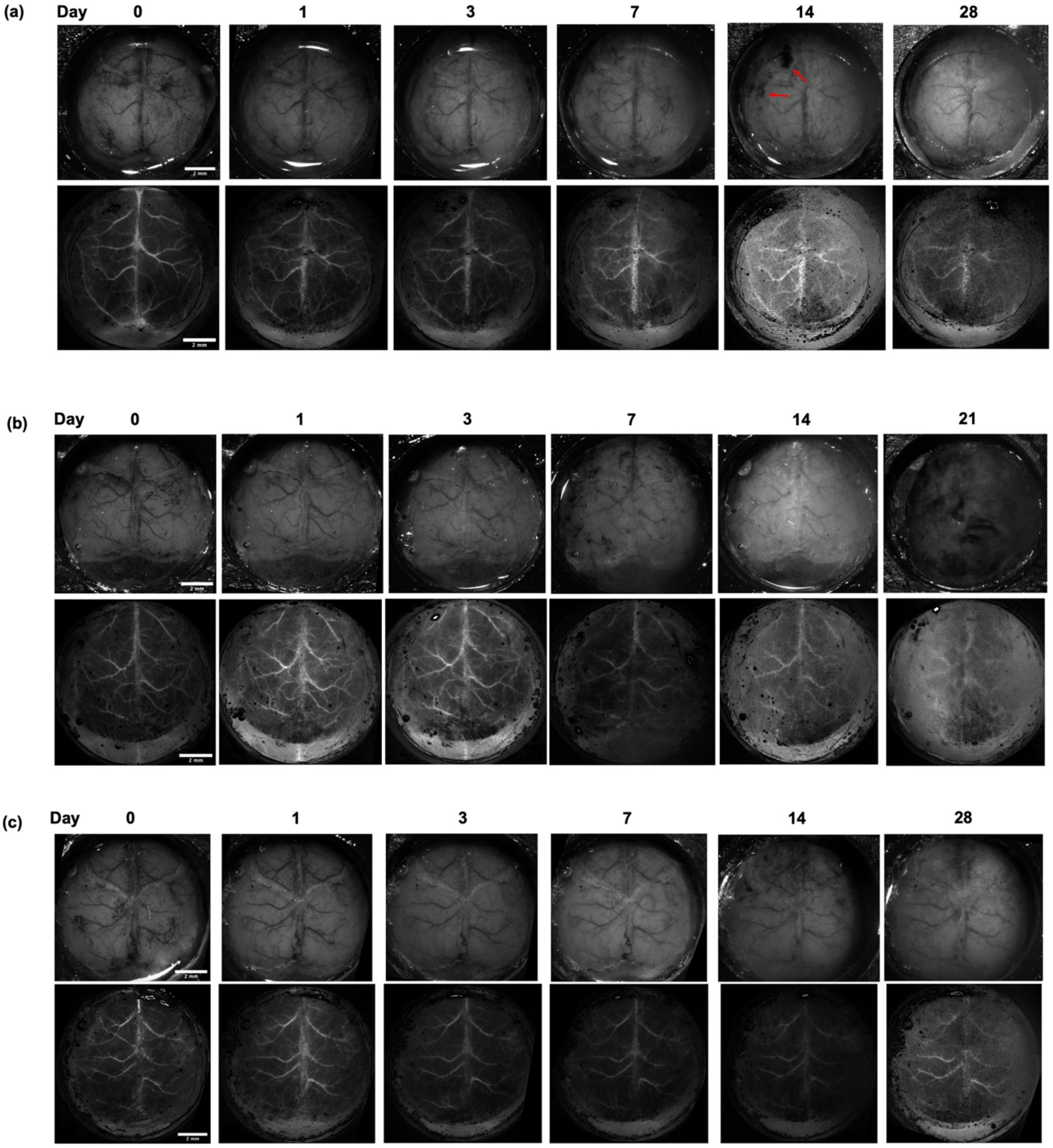

Figure S4 IOSI (top row) and LSCI (bottom row) images of Triple-S applied windows across time. **(a)** shows a window with regional inflammatory-like reaction (indicated by red arrows), and **(b)** shows full deterioration of the window, (c) shows a window with minimal deterioration after Triple-S application. Scale bar = 2 mm.
